# Real-world experience with ponatinib therapy in chronic phase chronic myeloid leukemia: impact of depth of response on survival and prior exposure to nilotinib on arterial occlusive events

**DOI:** 10.1038/s41408-023-00891-x

**Published:** 2023-08-11

**Authors:** Maymona G. Abdelmagid, Aref Al-Kali, Mark R. Litzow, Kebede H. Begna, William J. Hogan, Mirinal S. Patnaik, Shahrukh K. Hashmi, Michelle A. Elliott, Hassan Alkhateeb, Omer S. Karrar, Farah Fleti, Mohammed H. Elnayir, Candido E. Rivera, Hemant S. Murthy, James M. Foran, Mohamed A. Kharfan-Dabaja, Talha Badar, David S. Viswanatha, Kaaren K. Reichard, Naseema Gangat, Ayalew Tefferi

**Affiliations:** 1 Division of Hematology, Mayo Clinic, Rochester, MN USA; 2 Division of Hematology, Mayo Clinic, Jacksonville, FL USA; 3 Department of Laboratory Medicine and Pathology, Mayo Clinic, Rochester, MN USA

**Keywords:** Myeloproliferative disease, Myeloproliferative disease

## Abstract

We surveyed the performance of ponatinib, as salvage therapy, in a real-world setting of chronic phase chronic myeloid leukemia (CML-CP). Among 55 consecutive patients (median age 49 years) with relapsed/refractory CML-CP, 35 (64%) had failed ≥3 tyrosine kinase inhibitors (TKIs), 35 (64%) were pre-treated with nilotinib, and 14 (28%) harbored *ABL1*T315I. At start of ponatinib (median dose 30 mg/day), 40 patients were already in complete hematologic (CHR), 4 in complete cytogenetic (CCyR), 3 in major molecular (MMR) remission, while 8 had not achieved CHR (NR). Ponatinib improved the depth of response in 13 (33%), 3 (75%), 2 (66%), and 4 (50%) patients with CHR, CCyR, MMR, and NR, respectively (*p* = 0.02). At a median follow-up of 42 months, 13 (23%) deaths, 5 (9%) blast transformations, and 25 (45%) allogeneic transplants were recorded. Five/10-year post-ponatinib survival was 77%/58% with no significant difference when patients were stratified by allogeneic transplant (*p* = 0.94), ponatinib-induced deeper response (*p* = 0.28), or a post-ponatinib ≥CCyR vs CHR remission state (*p* = 0.25). *ABL1*T315I was detrimental to survival (*p* = 0.04) but did not appear to affect response. Prior exposure to nilotinib was associated with higher risk of arterial occlusive events (AOEs; 11% vs 0%; age-adjusted *p* = 0.04). Ponatinib starting/maintenance dose (45 vs 15 mg/day) did not influence either treatment response or AOEs. Our observations support the use of a lower starting/maintenance dose for ponatinib in relapsed/refractory CML-CP but a survival advantage for deeper responses was not apparent and treatment might not overcome the detrimental impact of *ABL1*T315I on survival. The association between prior exposure to nilotinib and a higher risk of post-ponatinib AOEs requires further validation.

## Introduction

Operational cure [1–3] is now a possibility in chronic phase chronic myeloid leukemia (CML-CP), with durable treatment-free remission (TFR) reported in approximately 40% of patients who had achieved imatinib-induced complete molecular remission (CMR) lasting for at least 2 years, and followed for a median of 6 years after treatment discontinuation [4]. CMR in CML-CP implies undetectable minimal residual disease (>5-log reduction in BCR::ABL1 transcript level) while deep molecular response (DMR) is operationally defined as a 4-log (MR^4^; BCR::ABL1/ABL1 ratio on the international scale ≤0.01%) or 4.5-log (MR^4.5^; ≤0.0032%) reduction in BCR::ABL1 transcript level whereas a 3-log reduction (≤0.1%) is considered major molecular remission (MMR) and a 2-log reduction (≤1%) complete cytogenetic remission (CCyR) [5].

TFRs in CML-CP have also been reported with other tyrosine kinase inhibitors (TKIs) and in the context of DMR at time of treatment discontiuation [3, 6]. In a recent multi-center prospective study of 173 patients with CML-CP receiving treatment with imatinib, dasatinib, nilotinib, or bosutinib, treatment was discontinued after documentation of continuous DMR for at least 2 years [6]; after a median follow-up of 41.6 months from time of treatment discontinuation, 65.5% stayed in MMR and the likelihood of molecular recurrence was lowest in the absence of detectable BCR::ABL1 at time of treatment discontinuation [6]. The importance of duration and depth of response, prior to TKI discontinuation, was confirmed in a more recent study of 199 CML-CP patients [3]; the 5-year TFR rate was 87–92% in patients with pre-TKI discontinuation DMR x ≥ 5 years vs 64% with DMR < 5 years; furthermore, virtually all patients with molecular relapse regained their pre-discontinuation remission status on re-treatment [3].

Despite the above-discussed advances in attaining deeper responses and now TFRs, neither “cure” nor MMR/DMR is necessarily a prerequisite for long-term survival in CML-CP, which might be possible by simply achieving TKI-induced CCyR [7]. The latter observation is practically relevant considering the consensus first-line drug of choice being imatinib, which might not necessarily be the most effective in rapidly inducing MMR/DMR [8–10], and yet arguably the safest and least expensive, among the currently FDA-approved TKIs for CML [11], the others being dasatinib, nilotinib, bosutinib, ponatinib, and asciminib. [9, 12–21] However, a substantial minority of patients with CML-CP are either intolerant or fail to achieve the desired response milestone with imatinib and, therefore, require treatment with an alternative TKI [22]. In this regard, our current preference as second-line drug of choice is dasatinib, based on its proven efficacy, even at a lower dose schedule [23], and the nature of associated side effects seen with the other TKIs, especially in terms of nilotinib-associated vascular complications, including arterial occlusive events (AOEs) [24, 25]. In our practice, we prefer to avoid nilotinib not only because of its well-described association with clinically overt AOEs but also because of its potential association with subclinical arterial anomalies [26].

Among currently approved TKIs, ponatinib and asciminib have distinguished themselves by their anti *BCR::ABL1*-T315I activity [27, 28]; however, as is the case with nilotinib, these drugs have also been associated with treatment-emergent AOEs [27, 28]. In a recent updated report on the use of ponatinib in two previously reported clinical trials, PACE and OPTIC, with a focus on patients with CML-CP, serious treatment-emergent adverse events and serious arterial occlusive events (AOEs) were documented in 63% and 18% in PACE and 34% and 4% in OPTIC [27]; however, among 94 patients assigned to the lower ponatinib dose (15 mg/day), in the OPTIC trial, only 3 (3.2%) experienced AOE during years 1 to 3, with otherwise similar 3-year overall survival to those initially assigned to the 45 or 30 mg/day dose level. The lower incidence of AOEs in patients receiving lower dose ponatinib has also been demonstrated in other real-world studies [29, 30]. In the current intra-institutional retrospective study, we wanted to assess the performance of ponatinib in a real-world setting, with focus on optimal dosing, correlation between response states and post-ponatinib survival, and the impact of prior exposure to nilotinib on ponatinib-associated vascular events.

## Methods

The current study was conducted under an institutional review board approved minimum risk protocol that allowed retrospective collection and analysis of data from patients with CP-CML, treated with ponatinib and were seen at the Mayo Clinic, USA (Rochester, Minnesota; Scottsdale, Arizona; Jacksonville, Florida). The International Consensus Classification (ICC) criteria were used for diagnosis and classification of CML [31]; diagnosis of CML-CP required absence of major-route chromosomal abnormalities (i.e., additional Ph1, trisomy 8, isochromosome 17q, trisomy 19, complex karyotype, abnormalities of 3q26.2), a myeloid blast percentage of <10% and lymphoid blast percentage of <5%, in the peripheral blood (PB) or bone marrow (BM), absence of extramedullary myeloid sarcoma, and PB basophil count of <20% [31]. Hematologic, cytogenetic, and molecular responses were assigned according to previously recommended guidelines [5]; complete hematologic remission (CHR) entailed normal blood cells and counts (absence of immature myeloid cells, leukocyte count <10 × 10(9)/L, basophils <5%, and platelet count <450 × 10(9)/L). Complete molecular remission (CMR) was considered in the absence of detectable minimal residual disease (>5-log reduction in BCR::ABL1 transcript level). Deep molecular remission (DMR) was defined as a 4-log (MR^4^; BCR::ABL1/ABL1 ratio on the international scale ≤0.01%) or 4.5-log (MR^4.5^; ≤0.0032%) reduction in BCR::ABL1 transcript level whereas a 3-log reduction (≤0.1%) was considered major molecular remission (MMR) and a 2-log reduction (≤1%) complete cytogenetic remission (CCyR) [5]. Laboratory data at time of diagnosis of CML-CP were available in 46 of the 55 study patients. Conventional statistical methods were applied using JMP Pro 16.0.0 software (SAS Institute, Cary, NC, USA); survival analysis was calculated from the time of initiation of ponatinib therapy to i) last follow-up or death, or ii) time of allogeneic hematopoietic stem cell transplant (AHSCT) or death or last follow-up; in the latter analysis, patients undergoing AHSCT were censored as being alive at time of their transplant.

## Results

### Clinical and laboratory data at time of initial diagnosis of CML-CP or prior to initiation of treatment with ponatinib

A total of 55 patients with relapsed/refractory or *ABL1*T315I-mutated CML-CP (median age at diagnosis 49 years, 51% females) received ponatinib between 2011 and 2022 and were evaluable for assessment of response and side effects; 4 patients received ponatinib post-AHSCT relapse. Clinical and laboratory information at time of initial diagnosis of CML-CP was available in 46 patients (Table 1). Cytogenetic details at time of initial diagnosis were available in 50 patients, all of whom displayed the classic Ph1 translocation with a median of 100% metaphase involved; 4 patients had a loss of Y chromosome, 1 patient had a deletion of 7q, and another had a 5q deletion. At the time of initiation of ponatinib (*N* = 55), median age was 54 years (range 30–85); age ≥60 years 22%; females 51%. Median number of TKIs received prior to ponatinib was 3, including 14 (25%) patients who had received 4 TKIs, 21 (38%) 3 TKIs, and 15 (27%) 2 TKIs. Prior TKIs included imatinib (*n* = 48; 87%), dasatinib (*n* = 46; 83%), nilotinib (*n* = 35; 64%), and bosutinib (*n* = 25; 45%). At the time of treatment start with ponatinib, 8 (14%) patients had not achieved CHR, 40 (72%) were in CHR, 4 (7%) in CCyR, and 3 (5%) in MMR; none were in DMR. *ABL1* mutation information was available in 50 patients prior to ponatinib start date: 23 (46%) patients harbored an *ABL1* mutation that included 14 (28%) with the T315I variant; other *ABL1* mutations included M244V, G250E, D276G, F317L, F359V, E255K, M351V, and E355A.

**Table 1:** Clinical and laboratory findings among 46 patients with chronic phase chronic myeloid leukemia, obtained at time of initial diagnosis, before subsequent progression into relapsed/refractory disease that required treatment with multiple tyrosine kinase inhibitors and ultimately with ponatinib.

Variables	All patients *N* = 46	Age <60 *N* = 35	Age ≥60 *N* = 11	*p* value	Patients with ≤2 prior TKIs *N* = 17	Patents with 3/4 prior TKIs *N* = 29	*p* value	Patients with prior Nilotinib *N* = 5	Patients without prior Nilotinib *N* = 41	*p* value
Age at diagnosis; median (range)	49 (24–83)	41 (24–59)	65 (62–83)	**<0.0001**	49 (30–83)	49 (24–78.5)	0.93	41.7 (26.5–64)	49 (24–83)	0.75
Females, *n* (%)	28 (52)	18 (51)	6 (54)	0.85	8 (47)	16 (55)	0.6	2 (40)	22 (53)	0.56
Leukocytes × 10^9^/L; median (range)	125 (13–498.7)	158 (13–498.7)	70 (14–378)	0.26	174 (33.7–498.7)	125 (13–366.8)	0.3	81 (47–181)	158 (13–498.7)	0.2
Absolute monocyte count ×10^9^/L; median (range)	3.1 (0.16–22)	4.6 (0.16–22)	2.8 (0.27–12.7)	0.37	7.4 (0.27–17.8)	2.7 (0.16–22)	**0.04**	6.4 (1.3–12.7)	3.1 (0.16–22)	0.8
Blood monocyte %	4 (0.8–13)	3.5 (1–8)	4 (0.8–13)	0.36	4.5 (0.8–7)	3 (1–13)	0.72	6 (3–13)	3 (0.8–8)	**0.02**
Absolute eosinophil count ×10^9^/L; median (range)	2.95 (0–22.8)	3.3 (0–22.8)	1.3 (0–22.7)	0.5	5.3 (0–22.8)	2.7 (0.1–9.5)	**0.01**	0.6 (0.4–5.4)	3.2 (0–22.8)	0.11
Blood eosinophil %	2.2 (0–9)	2.5 (0–9)	2 (0–6)	0.49	3 (0–9)	2 (0.8–6)	0.15	1 (1–3)	2.7 (0–9)	0.11
Absolute basophil count ×10^9^/L; median (range)	5.7 (0–47.8)	6.2 (0–47.8)	3.2 (0.3–22.7)	0.11	8 (1.3–47.8)	3.35 (0–25)	0.08	3.4 (0.8–3.7)	6 (0–47.8)	0.07
Blood basophil %	5 (1–18)	5 (1–18)	4 (1–17.2)	0.6	5 (2–18)	4 (1–15)	0.05	3 (1–8)	5 (1–18)	0.37
Absolute myelocyte count ×10^9^/L; median (range)	17.7 (0.26–110)	21 (0.26–110)	4.5 (0.5–83.2)	0.21	19.6 (2–102)	13.6 (0.26–110)	0.41	3.8 (2–8)	20.7 (0.26–110)	**0.01**
Blood myelocyte %	13.5 (2–34)	15 (2–34)	13 (2–28)	0.36	13 (2–34)	13.5 (2–30)	0.88	4.5 (2–17)	14 (2–34)	**0.03**
Blood blast count ×10^9^/L; median (range)	2 (0–34)	2.8 (0–18.5)	1 (0–34)	0.91	3.2 (0–34)	1.15 (0–16.7)	0.06	0.45 (0–3.6)	2.4 (0–34)	0.12
Blood blast %	2 (0–9)	2 (0–7)	1 (1–9)	0.8	3 (0–9)	1 (0–7)	**0.03**	1 (0–2)	2 (0–9)	0.23
Bone marrow blast %	1 (0–7)	1 (0–5)	1 (0–7)	0.55	1 (0–7)	1 (0–5)	0.62	2 (0–3)	1 (0–7)	0.92
Bone marrow reticulin fibrosis grade	0 (0–3)	0 (0–3)	0 (0–1)	**0.02**	0.5 (0–3)	0 (0–3)	**0.04**	0 (0–0)	0 (0–3)	0.06
Hemoglobin g/dL; median (range)	10.7 (5.6–15.5)	10.1 (5.6–15.5)	11.1 (7.4–15.1)	0.23	10.6 (7.2–14)	10.7 (5.6–15.5)	0.96	10.8 (8.6–12)	10.3 (5.6–15.5)	0.88
Platelets ×10^9^/L; median (range)	456.5 (110–2325)	398.5 (110–1308)	686 (180–2325)	**0.03**	507.5 (186–2243)	459 (110–2325)	0.85	383 (272–2243)	500.5 (110–2325)	0.24
BCR/ABL transcript:				0.08			0.33			**0.03**
Positive P120, *n* (%)	45 (97)	35 (100)	10 (91)		17 (100)	28 (96.5)		4 (80)	41 (100)	
Positive P190, *n* (%)	1 (2)	0 (0)	1 (9)		0 (0)	1 (3.4)		1 (20)	0 (0)	

Bold values indicates statistical significant *p* values.

### The impact of ponatinib based on pre-ponatinib remission states

Median starting dose of ponatinib was 30 mg/day (range, 10–45); the two major dose groups were 13 (24%) patients who were started and maintained on 15 mg/day and 18 (33%) patients who were started and maintained on 45 mg/day. Among all evaluable patients (*n* = 51), median ponatinib initial dose (range) was 30 mg (10–45); among 13 evaluable cases T315I mutation, median ponatinib initial dose (range) was 45 mg (15–45); among 33 evaluable cases with non-T315I *ABL1* mutation, median ponatinib initial dose (range) was 30 mg (10–45). As mentioned before, the pre-ponatinib remission states included MMR (*N* = 3; 5%), CCyR (*N* = 4; 7%), CHR (*N* = 40; 73%), and NR (*N* = 8; 14%). The best post-ponatinib remission states included DMR (*N* = 10; 18%), MMR (*N* = 8; 15%) CCyR (*N* = 4; 7%), CHR (*N* = 29; 53%), and NR (*N* = 4; 7%). Ponatinib therapy improved the response level in 4 (50%) of 8 patients with NR, 13 (33%) of 40 with prior CHR, 3 (75%) of 4 with prior CCyR, and 2 (66%) of 3 with prior MMR (Table 2). The likelihood of ponatinib-induced improvement in response was significantly higher in patients with pre-ponatinib remission state of CCyR (86%) vs CHR (32%) vs NR (50%; *p* = 0.02) but it was not affected by the number of TKIs received or the presence or absence of T315I *ABL1* mutation (36% vs 42%, respectively; *p* = 0.69). There was no significant correlation between ponatinib dose and response; 9 (50%) of 18 on the 45 mg/day start/maintained group vs 4 (31%) of 13 on the 15 mg/day start/maintained groups experienced a ponatinib-induced response upgrade (*p* = 0.86); DMR was attained in 2 (11%) patients on the 45 mg/day group and in 4 (31%) patients on the 15 mg/day group.

**Table 2:** Best response achieved by ponatinib therapy among 55 patients with relapsed/refractory/T315I chronic myeloid leukemia in chronic phase, stratified by pre-ponatinib remission states.

Best response on Ponatinib	Patients with no Remission *N* = 8	Patients in CHR *N* = 40	Patients in CCyR *N* = 4	Patients in MMR *N* = 3
Achieved complete hematologic remission, *n* (%)	1 (12.5)	N/A	N/A	N/A
Achieved complete cytogenetic remission, *n* (%)	1 (12.5)	3 (7)	N/A	N/A
Achieved major molecular remission, *n* (%)	2 (25)	5 (12)	0 (0)	N/A
Achieved deep molecular remission, *n* (%)	0 (0)	5 (12)	3 (75)	2 (66)
Lost previous response, *n* (%)	N/A	0 (0)	1 (25)	0 (0)

### Ponatinib treatment-emergent side effects

Ponatinib was discontinued in 41 (75%) patients; median duration of treatment in patients still on treatment at the time of this writing (*n* = 14) was 39 months (range, 10–118) and 5 months (range, 0–46) in those in whom treatment was discontinued. The reasons for ponatinib treatment discontinuation included side effects/toxicity (*n* = 25; 60%), suboptimal response (*n* = 9; 21%), planned AHSCT (*n* = 7; 17%), and medication nonadherence in 2 (4%). Table 3 outlines details of ponatinib treatment-emergent side effects with the most frequent being cytopenias in 17 (30%), skin rash in 11 (20%), muscle/joint pain in 10 (18%), fatigue in 6 (11%), increased transaminases in 6 (11%), clinical pancreatitis in 5 (9%), headache in 5 (9%), nausea and vomiting in 5 (9%), and AOEs in 4 (7%). Comparison of side effects in the presence or absence of prior exposure to nilotinib therapy revealed a significant difference in AOEs, which was more frequent in patients exposed to nilotinib (11% vs 0% in patients not exposed to nilotinib; age-adjusted *p* = 0.04); AOEs also correlated with age ≥60 years (*p* = 0.01) but significance was lost during multivariable analysis that included age, ponatinib dose, and ponatinib treatment duration while the age-independent association with previous exposure to nilotinib therapy was sustained (*p* = 0.04). By contrast, neither the ponatinib dose range nor the number of TKIs received prior to ponatinib therapy appear to influence the post-ponatinib occurrence of AOEs (Table 3). Among the 4 patients with AOEs observed on ponatinib (ages 39, 68, 71 and 82 years and initial ponatinib doses 45, 15, 45, 15 mg, respectively), all four were previously exposed to nilotinib. The corresponding AOEs were carotid artery stenosis/stroke, coronary artery disease, unstable angina, and arterial thrombosis. The duration range of treatment for ponatinib was 5–46 months and for nilotinib 3–36 months.

**Table 3:** Ponatinib side effects/toxicity profile for 55 patients with relapsed/refractory chronic phase chronic myeloid leukemia stratified by age and prior tyrosine kinase inhibitors.

Side effects	All patients *N* = 55	Age <60 *N* = 43	Age ≥60 *N* = 12	*p* value	Patients with ≤2 prior TKIs *N* = 20	Patents with 3/4 prior TKIs *N* = 35	*p* value	Patients with prior Nilotinib *N* = 35	Patients without prior Nilotinib *N* = 20	*p* value
Arterial vascular events, *n* (%)	4 (7%)	1 (2%)	3 (25%)	**0.01**	1 (5%)	3 (8%)	0.6	4 (11%)	0 (0%)	**0.05**
Arterial thrombosis	1 (1.8%)	0 (0%)	1 (8%)	0.07	0 (0%)	1 (2%)	0.33	1 (3%)	0 (0%)	0.33
Angina	1 (1.8%)	0 (0%)	1 (8%)	0.07	0 (0%)	1 (2%)	0.33	1 (3%)	0 (0%)	0.33
Acute coronary artery	1 (1.8%)	0 (0%)	1 (8%)	0.07	0 (0%)	1 (2%)	0.33	1 (3%)	0 (0%)	0.33
Carotid artery stenosis/stroke	1 (1.8%)	1 (2%)	0 (0%)	0.48	1 (5%)	0 (0%)	0.15	1 (3%)	0 (0%)	0.33
Cytopenia, *n* (%)	17 (30%)	12 (27%)	5 (41%)	0.37	5 (25%)	12 (34%)	0.46	12 (34%)	5 (25%)	0.47
Skin rash, *n* (%)	11 (20%)	8 (18%)	3 (25%)	0.63	7 (35%)	4 (11%)	**0.03**	5 (14%)	6 (30%)	0.16
Muscle and joint pain, *n* (%)	10 (18%)	6 (14%)	4 (33%)	0.14	7 (35%)	3 (8%)	**0.01**	6 (17%)	4 (20%)	0.8
Fatigue, *n* (%)	6 (11%)	3 (6%)	3 (25%)	0.10	2 (10%)	4 (11%)	0.86	3 (8%)	3 (15%)	0.47
Transaminitis, *n* (%)	6 (11%)	5 (11%)	1 (8%)	0.73	1 (5%)	5 (14%)	0.26	3 (8%)	3 (15%)	0.47
Pancreatitis, *n* (%)	5 (9%)	5 (11%)	0 (0%)	0.10	3 (15%)	2 (5%)	0.26	2 (5%)	3 (15%)	0.26
Headache, *n* (%)	5 (9%)	4 (9%)	1 (8%)	0.91	3 (15%)	2 (5%)	0.26	3 (8%)	2 (10%)	0.86
Nausea and vomiting, *n* (%)	5 (9%)	4 (9%)	1 (8%)	0.91	2 (10%)	3 (8%)	0.86	4 (11%)	1 (5%)	0.4
Hepatotoxicity, *n* (%)	4 (7%)	4 (9%)	0 (0%)	0.15	3 (15%)	1 (2%)	0.10	1 (3%)	3 (15%)	0.1
Palpitation, *n* (%)	4 (7%)	2 (4%)	2 (16%)	0.19	1 (5%)	3 (8%)	0.61	3 (8%)	1 (5%)	0.61
Hypertension, *n* (%)	3 (5%)	3 (6%)	0 (0%)	0.21	1 (5%)	2 (5%)	0.91	2 (5%)	1 (5%)	0.9
Ejection fraction reduction, *n* (%)	2 (3%)	1 (2%)	1 (8%)	0.37	0 (0%)	2 (5%)	0.17	2 (5%)	0 (0%)	0.17
Elevated bilirubin, *n* (%)	2 (3%)	2 (4%)	0 (0%)	0.31	1 (5%)	1 (2%)	0.68	1 (3%)	1 (5%)	0.68
Elevated amylase/lipase	2 (3%)	2 (4%)	0 (0%)	0.31	1 (5%)	1 (2%)	0.68	2 (5%)	0 (0%)	0.17
Neuropathy, *n* (%)	1 (1.8%)	1 (2%)	0 (0%)	0.48	0 (0%)	1 (2%)	0.33	1 (3%)	0 (0%)	0.33
Eye discomfort, *n* (%)	1 (1.8%)	0 (0%)	1 (8%)	0.07	1 (5%)	0 (0%)	0.15	0 (0%)	1 (5%)	0.15
Congestive heart failure, *n* (%)	1 (1.8%)	1 (2%)	0 (0%)	0.48	0 (0%)	1 (2%)	0.33	1 (3%)	0 (0%)	0.33
Diarrhea, *n* (%)	1 (1.8%)	0 (0%)	1 (8%)	0.07	1 (5%)	0 (0%)	0.15	0 (0%)	1 (5%)	0.15

Bold values indicates statistical significant *p* values.

### Post-ponatinib survival analysis

**Fig. 1:**
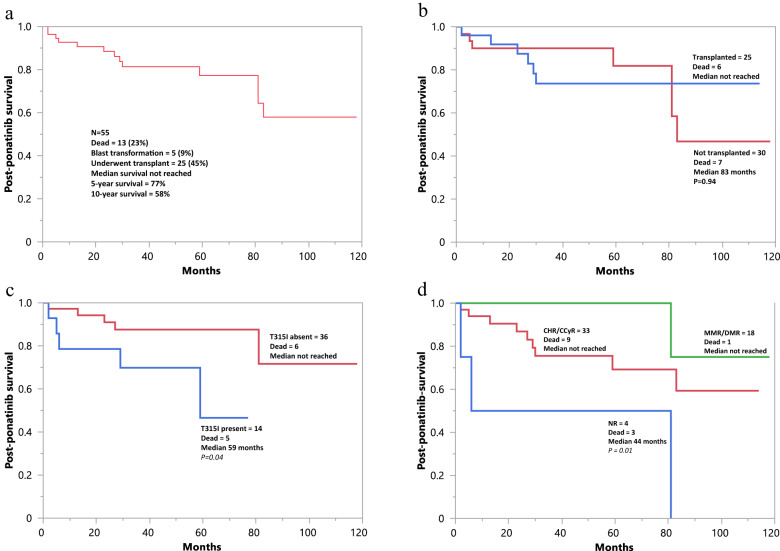
Post-ponatinib survival data. Post-ponatinib survival data in 55 patients with relapsed/refractory/T315I chronic phase chronic myeloid leukemia (**a**); stratified by allogeneic hematopoietic stem cell transplantation (**b**); stratified by presence or absence of the *ABL1-T315I* mutation (**c**); and stratified by best response achieved with ponatinib treatment (**d**). NR no remission, CHR complete hematologic remission, CCyR complete cytogenetic remission, MMR major molecular remission, DMR deep molecular remission.

**Fig. 2:**
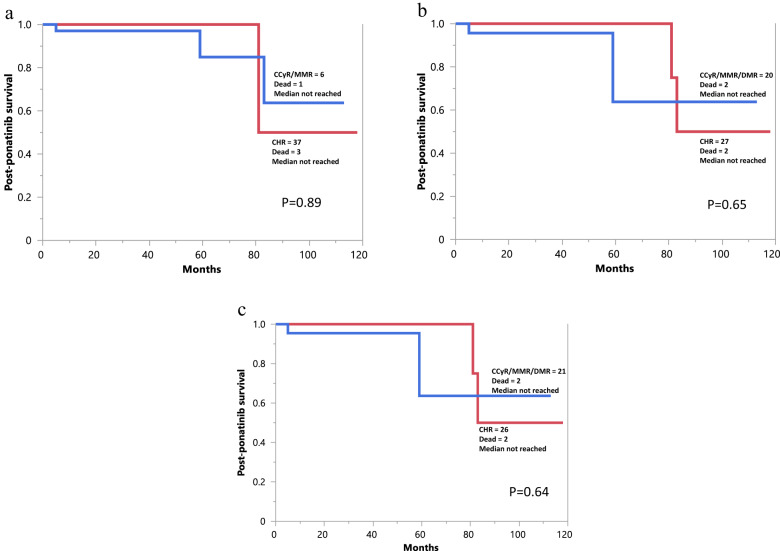
Post-ponatinib survival data. Post-ponatinib survival data of patients with relapsed/refractory/T315I chronic phase chronic myeloid leukemia, calculated after censoring patients who received allogeneic hematopoietic stem cell transplantation and stratified by pre- (**a**; *N* = 43), post- (**b**; *N* = 47), or best pre- or post-ponatinib (**c**; *N* = 47) remission status. Eight patients were excluded from the survival analysis, including four patients who received ponatinib after post-transplant relapse of CML and another four who failed to achieve CHR (survival comparisons were between CHR and higher levels of response). CHR complete hematologic remission, CCyR complete cytogenetic remission, MMR major molecular remission, DMR deep molecular remission.

At a median follow-up of 8 years from diagnosis and 42 months (range 2–118) from initiation of ponatinib therapy, 13 (23%) deaths, 5 (9%) blastic transformations, and 25 (45%) AHSCTs were documented. Median post-ponatinib survival was not reached with 5- and 10-year survival rates of 77% and 58%, respectively (Fig. 1a). Figure 1b illustrates survival data stratified by AHSCT (*p* = 0.94), Fig. 1c by presence or absence of T315I *ABL1* mutation (*p* = 0.04), and Fig. 1d by post-ponatinib remission state (*p* = 0.01). In multivariable analysis for overall survival, which included age and AHSCT, the significant impact of T315I (HR 4.5. 95% CI 1.2–17.2; *p* = 0.01) and NR vs MMR/DMR (HR 18, 95% CI 1.8–183.2; *p* < 0.01) were confirmed; age and AHSCT were not significant. A broader analysis of post-ponatinib survival did not identify additional risk factors for survival. We next conducted survival analysis after accounting for AHSCT by censoring transplanted patients at time of their transplant; the overall results were unchanged and showed similar survival data in patients with best documented response of CHR vs CCyR/MMR/DMR, assigned before (Fig. 2a; *p* = 0.89), after (Fig. 1b; *p* = 0.65), or either before or after (Fig. 1c; *p* = 0.64).

### Influence of ponatinib-induced response improvement on survival and other events

Among 8 patients who were not in CHR at time of ponatinib therapy, 4 achieved MMR (*n* = 2), CCyR (*n* = 1) or CHR (*n* = 1). Among these 4 responders, 2 (50%) have since died (compared to 3 of the 4 non-responders; *p* = 0.46), one (25%) progressed into blast transformation (compared to 2 of 4 non-responders; *p* = 0.46), one (25%) harbored *ABL1* T315I (compared to 2 of 4 non-responders; *p* = 0.21); post-ponatinib survival in the 4 non-responders was 81 months (dead), 66 months (with AHSCT, alive), 6 months (dead), and 2 months (dead), and in the 4 responders 41 months (alive), 30 months (with AHSCT; dead), 28 months (alive), and 13 months (with AHSCT; dead). Among the forty patients in CHR at time of ponatinib treatment start, 13 (33%) experienced deeper responses (Table 3). Among these 13 responders, one (8%) has since died (compared to 6 of 27 non-responders; *p* = 0.23), one (8%) progressed into blast transformation (compared to one of 27 non-responders; *p* = 0.6), 5 (39%) underwent AHSCT (compared to 16 of 27 non-responders; *p* = 0.21), 3 (27%) harbored T315I (compared to 7 of 25 non-responders; *p* = 0.96); overall survival was similar between responders and non-responders (*p* = 0.24). Among the 7 patients with CCyR or MMR at start of treatment with ponatinib, 5 (71%) experienced deeper responses (Table 3). Among these 5 responders, one (20%) has since died (compared to 0 of 2 non-responders; *p* = 0.39), none progressed into blast transformation (compared to none of the non-responders; *p* = NS), one (20%) harbored T315I (compared none of the 2 non-responders; *p* = 0.39), none received AHSCT (compared to one of the 2 non-responders; *p* = 0.08).

### Outcome in patients with T315I ABL1 mutation, who underwent AHSCT or blast transformation

A total of 50 patients had *ABL1* mutation information prior to ponatinib therapy; 14 (28%) expressed T315I. Comparison of patients with or without T315I revealed no significant differences in terms of likelihood for ponatinib-induced response upgrade (*p* = 0.7), pre-ponatinib remission status (*p* = 0.5), treatment-emergent AOEs (*p* = 0.9) or pancreatitis (*p* = 0.11); similarly, the presence of non-T315I *ABL1* mutations did not affect either ponatinib response, incidence of AOEs (3 of 4 incidents occurred in patients without T315I mutation), or post-ponatinib survival (data not shown because of small numbers). However, in both univariate and multivariable analysis, the presence of T315I was associated with inferior survival (Fig. 1c). A total of 25 patients underwent AHSCT; transplanted patients were more likely to be younger (*p* = 0.045) but the two groups displayed similar survival, although the survival curve for AHSCT appeared to plateau after 2 years (Fig. 1b). A total of 5 patients, all aged <60 years, experienced blast transformation; 4 (80%) of the 5 patients have since died (*p* = 0.005); patients with blast transformation were more likely to have been in either NR (3 patients) or CHR (2 patients) prior to initiation of ponatinib therapy (*p* = 0.03); of these 5 patients with blast transformation, the post-ponatinib remission state was NR in 2 patients, CHR in one patient, CCyR in one patient, and MMR in the 5^th^ patient. Patients with or without blast transformation were otherwise similar in other aspects, including T315I expression (33%; *p* = 0.83).

## Discussion

The current real-world study confirms observations from clinical trials, on the activity and toxicity pattern of ponatinib in the treatment of relapsed/refractory CML. We were most encouraged by the relatively low frequency of ponatinib treatment-emergent AOEs, especially in the absence of previous exposure to nilotinib; none of our patients without such exposure developed post-ponatinib cardiovascular complications while the incidence was 11% in those previously exposed to nilotinib. The association between previous exposure to nilotinib and post-ponatinib AOEs in the current study was independent of age, ponatinib dose, or treatment duration; accurate information on cardiovascular risk factors was not available to assess potential confounding. By contrast, the number of TKIs received prior to ponatinib therapy did not appear to influence either response or toxicity patterns. These observations support our current practice of avoiding nilotinib as first- or second-line TKI of choice, in the treatment of CML-CP, especially considering the availability of other second generation TKIs with similar efficacy but devoid of vascular toxicity. The association of nilotinib therapy in CML with AOEs was first described in 2011 [24] and subsequently confirmed by a larger study that reported an incidence rate of 29.4% (compared to <5% in age-matched controls), after a median follow-up of 2 years [32]. In another study of 220 patients with CML-CP receiving nilotinib therapy, vascular events were reported in 12%, with an incidence of 4.1 events per 100 patient-years and risk factors identified included older age and dyslipidemia [33]. While these reports were focused on overt arterial events, the possibility of nilotinib-associated subclinical endothelial injury was raised by a more recent study that described the presence of ultrasound arterial anomalies (plaques, stenosis, occlusion) in 25 (34%) of 75 nilotinib-treated patients, with the carotid bulb being the most frequently involved territory [26]; the reported anomalies were more frequent in older patients and in those with history of hypertension and cardiovascular risk factors [26]. On the other hand, our current practice of using dasatinib as our second-line TKI of choice in CML-CP, with imatinib being our first-line TKI of choice, is further supported by recent demonstration of the possibility of using lower doses of dasatinib, in order to mitigate drug-associated side effects, without compromising efficacy [15, 23].

Observations from the current study are also supportive of our current practice of using ponatinib as the preferred third-line TKI of choice. The prevalence of AOEs in the current real-world study was very low, in the absence of prior treatment with nilotinib. It is possible that our preference of using lower doses of the drug might have contributed to this favorable observation, although we saw no correlation between AOE and ponatinib dose, in the current study. These observations are consistent with recent clinical trial updates on long-term efficacy and toxicity of ponatinib in both chronic and advanced phase CML [27]. The particular update included patients with Ph1-positive acute lymphoblastic leukemia (Ph+ ALL) and CML (PACE and OPTIC trials) [27]. In the OPTIC trial, patients with CP-CML and resistance to ≥2 prior TKIs or *ABL1* T315I mutation were initially dosed at ponatinib 45 or 30 mg/day and their dose reduced to 15 mg/day at time of CCyR [27]. Among patients with CML-CP in the PACE (*n* = 257) and OPTIC (*n* = 93) trials starting with ponatinib 45 mg/day, the 2-year CCyR and overall survival rates were 46% and 85%, respectively, in PACE and 57% and 91%, in OPTIC, respectively, while the incidence of AOEs were 18% in PACE and 4% in OPTIC [27]. The latter figure is close to what was seen in the current study whose design was more in line with that of OPTIC, as well as other retrospective studies where lower doses of ponatinib were used [29, 30]. Our preference of starting with the lower dose of ponatinib was intended to minimize clinically overt and subclinical vascular injury, and does not preclude upward dose titration guided by close monitoring of response. Several other studies have also looked into the experience with ponatinib [34–36].

Another important observation from the current study concerns the lack of significant correlation between post-ponatinib survival and a remission state beyond CHR, regardless of whether or not the response level was achieved before or after treatment with ponatinib. Furthermore, ponatinib-induced maintenance or achievement of a response level ≥CHR did not appear to depend on the dose of ponatinib administered and was similar in the context of 15 or 45 mg/day start-maintenance doses. We find these observations practically relevant in terms of decision making on starting dose of ponatinib and whether or not it is necessary to increase the dose in search of DMR. A conservative approach on utilizing the lowest dose of the drug that secures durable CCyR is currently our preference. Obviously, our stance is subject to change based on additional information and we are fully cognizant of the limitations of a retrospective study of relatively small sample size with an even smaller number of informative cases.

Finally, we would like to address our position on the use of bosutinib, asciminib and AHSCT, in CML-CP. In general, our enthusiasm for bosutinib has been dampened by its frequent association with diarrhea and the results of a recent phase-3 study (ASCEMBL) that showed superiority of asciminib over bosutinib, both in terms of both efficacy and toxicity [37]; incidentally, the incidence of AOEs was 1.3% with bosutinib and 5.1% with asciminib, the latter of which is not trivial. The mechanism of action for asciminib involves BCR::ABL1 inhibition through allosteric inhibition targeting the myristoyl pocket of ABL1, as opposed to direct inhibition of the ATP-binding site of ABL1, which is the case with other TKIs [28]. Asciminib is currently FDA approved for use in CML-CP, in the presence of T315I (approved dose 200 mg BID, which is ridiculously expensive) or after failing ≥2 TKIs (approved dose 40 mg BID or 80 mg QD). Noteworthy side effects of asciminib include myelosuppression including thrombocytopenia and lipase elevation that is often not associated with clinical pancreatitis [28].

Without a controlled study, it is impossible to accurately assess the value and drawbacks of asciminib vs ponatinib. Data are equally scarce on the activity of asciminib in ponatinib-exposed patients with CML-CP, and vice versa [38, 39]; the same holds true for emerging new therapies that target T315I, including olverembatinib [40]. In the meantime, considering the longer experience with ponatinib and possibility of using a lower dose of the drug support our preference to use ponatinib as the preferred TKI after failure of imatinib and dasatinib. In regards to AHSCT, in CML-CP, the question is not whether it works but when it ought to be offered [41]; in other words, when does one determine that additional treatment attempts with yet another TKI is more likely to compromise AHSCT eligibility rather than result in net survival gain [42]. Our observations from the current study, in this regard, suggest that it might be safe to defer transplant as long as durable CHR is secured; yet, patients should be referred to transplant centers for evaluation and to identify suitable HLA compatible donors, to be able to proceed with the AHSCT as soon as it becomes indicated. The more difficult question to tackle is whether or not it is always necessary to use a more potent, more expensive, and, often, more dangerous TKI, in order to improve upon a CCyR, or even a CHR, which is often achieved by a first- or second-generation TKI, and might be adequate enough to secure long-term survival [7]. Finally, we are fully cognizant of the limitations of the current retrospective study including its relatively small sample size, heterogeneity in patients and treatment strategies, the lack of accurate information on cardiovascular risk factors, and variable monitoring time points.

## Data Availability

Through corresponding author.
